# Prior probability biases perceptual choices by modulating the accumulation rate, rather than the baseline, of decision evidence

**DOI:** 10.1162/imag_a_00338

**Published:** 2024-11-18

**Authors:** Jessica A. Diaz, M. Andrea Pisauro, Ioannis Delis, Marios G. Philiastides

**Affiliations:** School of Social Sciences, College of Psychology Birmingham City University, Birmingham, United Kingdom; School of Psychology and Neuroscience University of Glasgow, Glasgow, United Kingdom; Centre for Human Brain Health, University of Birmingham, Birmingham, United Kingdom; School of Psychology, University of Plymouth, Plymouth, United Kingdom; School of Biomedical Sciences University of Leeds, Leeds, United Kingdom

**Keywords:** prior probability, decision making, electroencephalography, hierarchical drift diffusion model, neurally-informed modeling

## Abstract

The prior probability of an upcoming stimulus has been shown to influence the formation of perceptual decisions. Computationally, these effects have typically been attributed to changes in the starting point (i.e., baseline) of evidence accumulation in sequential sampling models. More recently, it has also been proposed that prior probability might additionally lead to changes in the rate of evidence accumulation. Here, we introduce a neurally-informed behavioural modelling approach to understand whether prior probability influences the starting point, the rate of evidence accumulation or both. To this end, we employ a well-established visual object categorisation task for which two neural components underpinning participants’ choices have been characterised using single-trial analysis of the electroencephalogram. These components are reliable measures of trial-by-trial variability in the quality of the relevant decision evidence, which we use to constrain the estimation of a hierarchical drift diffusion model of perceptual choice. We find that, unlike previous computational accounts, constraining the model with the endogenous variability in the relevant decision evidence results in prior probability effects being explained primarily by changes in the rate of evidence accumulation rather than changes in the starting point or a combination of both. Ultimately, our neurally-informed modelling approach helps disambiguate the mechanistic effect of prior probability on perceptual decision formation, suggesting that prior probability biases primarily the interpretation of sensory evidence towards the most likely stimulus.

## 1 Introduction

In naturalistic environments, perceptual stimuli do not occur in isolation or independently of the context in which they are embedded. For example, the presence of roads and street signs increases the probability that there will also be vehicles in the scene. Likewise, the sound of your front door opening is often followed by the sight or sound of a familiar person. These examples highlight the importance of prior probability (i.e., the probability of a specific perceptual stimulus emerging in the scene) for the enhancement of the efficiency of perceptual processing (de Lange et al., 2018;Oliva & Torralba, 2007).

More specifically, a number of studies have shown that objects are identified more accurately when they are in familiar scenes and consistent backgrounds, for example, a toaster is recognised faster when seen in a kitchen (Auckland et al., 2007;Turk-Browne et al., 2010).

Similarly, temporal statistical relationships between perceptual stimuli have been used to predict upcoming stimuli across domains (Coull & Nobre, 2008;Leonard et al., 2016) and appear to arise very early in development (Fiser & Aslin, 2002;Nobre et al., 2007). Although it is well established behaviourally that prior probability affects how perceptual decisions are made, the neurobiological mechanisms underlying the role of prior probability remain less well understood (Summerfield & De Lange, 2014).

The conventional view in this area postulates that prior probability affects perceptual decision making by inducing changes in the baseline activity of regions involved in the encoding of the relevant decision evidence. Consistent with this view animal electrophysiology and human brain imaging, studies have argued that prior probability could affect baseline activity in category-specific sensory areas (Albright, 2012;Charlton & Goris, 2024;Esterman & Yantis, 2010;A. M. Puri et al., 2009) as well as induce activation of stimulus templates reflecting prior expectations (Domenech & Dreher, 2010;Feuerriegel et al., 2021;González-García & He, 2021;Kok et al., 2013,^2014^,^2017^). Similarly, studies have shown that temporal expectations can influence baseline activity in anticipation of an upcoming stimulus (Aitken et al., 2020;Barne et al., 2022;Basso & Wurtz, 1998;de Lange et al., 2013). A complementary account posits that prior probability will affect the baseline of the motor areas planning the action to report the appropriate choice, give the response to the stimulus, rather than that of the area encoding it. Consistently, studies have found effects in motor areas resulting from the presentation of prior information about the stimulus (de Lange et al., 2013;Feuerriegel et al., 2021;Gold & Stocker, 2017;Kelly et al., 2021).

More recently, studies have also argued that prior probability could additionally affect the actual interpretation of the relevant sensory evidence (i.e., how the evidence is used for the decision in higher-level brain areas). Specifically, animal electrophysiology studies have reported increases in firing rate in decision-related areas (e.g., lateral intraparietal area) with increasing match between the stimuli expected on the basis of a prior probability cue and the actual observed stimuli (Hanks et al., 2011). Conversely, human brain imaging studies have shown that unexpected stimuli with respect to prior probability increased fMRI activity in areas of the inferior temporal cortex during a perceptual categorisation task (K. Dunovan & Wheeler, 2018) and that temporal expectations modulated the signal-to-noise gain of visual information processing during the decision process itself, consistent with a pre-sensory prediction signal that scaled with probability (Cravo et al., 2013).

Accumulation-to-bound models (Ratcliff & McKoon, 2008) have similarly produced mixed results on how behavioural effects of prior probability could be explained at a mechanistic level. In line with the conventional view, some studies have reported changes primarily in the starting point of evidence accumulation, which moves the decision variable closer to one of the alternative decision boundaries (Feuerriegel et al., 2021;Forstmann et al., 2010;Leite & Ratcliff, 2011;Mulder et al., 2012). More recent studies have proposed that prior probability could additionally alter the quality of the post-sensory evidence entering the decision (i.e., drift rate in evidence accumulation), in a range of perceptual discrimination tasks with explicit (K. Dunovan & Wheeler, 2018;Kelly et al., 2021) as well as implicit (i.e., altering the temporal expectations, that is, information about when a stimulus is meant to appear rather than which one) (Cravo et al., 2013;Rohenkohl et al., 2012) manipulations of prior probability.

These results highlight that even though standard (behaviour-only) DDMs can, in principle, disambiguate sufficiently distinct RT distributions, there can be situations where different computational models could explain behaviour equally well even when the underlying (mechanistic) assumptions and/or the sampled distribution of reaction times are different (Lerche & Voss, 2016;Teichert et al., 2016). This ultimately hinders selection of the best model and, consequently, poses a difficulty in understanding the mechanistic origin of the observed behaviour and how this generalises across specific tasks (van Ravenzwaaij et al., 2017).

To address this problem, recent developments have also proposed the incorporation of relevant neural signatures into the estimation of these models (i.e., neurally-informed models) (Dully et al., 2018;Williams et al., 2021). This approach has the potential of constraining the model parameters to yield a more parsimonious and biologically-plausible explanation of decision formation, thereby facilitating a more in-depth understanding of the neural implementation of the underlying processes (Gläscher & O’Doherty, 2010;Turner et al., 2015). Here, we employ such a neurally-informed modelling approach to arbitrate between the competing accounts of whether prior probability influences the starting point, the rate of evidence accumulation or both, in the context of a classic perceptual decision-making task.

In this endeavour, we capitalise on a well-established visual object categorisation task (Philiastides et al., 2006;Philiastides & Sajda, 2006) in which trial-by-trial electrophysiological (EEG) activity reflecting the quality of the evidence entering the decision process (depending on both the noise of the stimulus and that of its neural representations at different stages of perceptual processing) could be reliably dissociated using multivariate pattern analysis (Diaz et al., 2017;Philiastides & Sajda, 2007;Ratcliff et al., 2009) and use it to model the choice and reaction time data (i.e., choice-RT data). More specifically, we integrate these trial-wise neural representations as additional parameter predictors of drift rate in a hierarchical drift-diffusion model (HDDM) to directly inform model selection and obtain a mechanistic understanding of the influence of prior probability on perceptual decision making. In contrast to traditional (behaviour-only) modelling, our results indicate that, in a simple visual categorisation task, prior probability primarily leads to changes in the quality of post-sensory evidence entering the decision (i.e., drift rate changes) rather than changes in baseline activation of the relevant decision variables. We also suggest that neurally-informed cognitive modelling can help disambiguate between competing hypotheses on the mechanistic underpinnings of behavioural effects.

## 2 Materials and Methods

### 2.1 Participants

Sixteen human participants (5 men and 11 women, age range 21–35 years) took part in this study. Each participant completed three different sessions (across three consecutive days; 3 x 16 = 42 experimental sessions). All were right-handed, reported normal vision and no history of neuro- logical problems. The study was approved by the College of Science and Engineering Ethics Committee at the University of Glasgow (CSE01353), and informed consent was obtained from all participants.

### 2.2 Stimuli

The stimuli were selected from those described in (Philiastides & Sajda, 2006,^2007^) to have noisy images of either cars or faces that subjects had to discriminate. The stimulus set was generated as follows. A set of 20 images of faces were selected from the Face Database of the Max Planck Institute of Biological Cybernetics (Blanz & Vetter, 1999;Troje & Bülthoff, 1996), and a set of 20 images of cars was sourced from the web. Each image was 512 x 512 pixels, with 8 bits per pixel, and there were equal numbers of frontal and side (up to 45°) views. All images were placed on a uniform grey background and were equated for spatial frequency, luminance and contrast. They all had identical magnitude spectra and their corresponding phase spectra were manipulated using the weighted mean phase (Dakin et al., 2002) technique to generate a set of images characterised by their percentage phase coherence. For each image, a set of 13 noisy variants were created. The noise levels were described in terms of*coherence*and ranged uniformly from 20% (lowest coherence, highest noise) to 50% (highest coherence, lowest noise level). As such, in this stimulus set there were a total of 2 x 20 x 13 = 520 images. We selected two levels of sensory evidence for this study (32.5% and 37.5% phase coherence), based on previous studies (Philiastides et al., 2006;Philiastides & Sajda, 2006).

A Dell Precision Workstation (Intel Core 2 Quad) running Windows 7 (64 bit) with an ATI FirePro 2270 graphics card and PsychoPy2 (Version 1.8) presentation software (Brooks, 2019;Peirce, 2007) controlled the stimulus display. Images were presented on a Dell 2001FP TFT monitor (resolution, 1,600 x 1,200 pixels; refresh rate, 60 Hz).

### 2.3 Behavioural task

Participants were presented on each trial with a noisy face or car stimulus as described above and performed a two-alternative categorisation task whereby they classified each image as either a face or a car as quickly and as accurately as possible. Participants’ choice and reaction time were recorded on each trial. Participants sat a distance of 75 cm from the computer monitor so that each image was around 6 x 6° of visual angle. At the start of each trial, a text-based cue was displayed for a duration of 750 ms. There were three different cues. The first indicates a 70% probability of face and 30% probability of car (70 F). The second indicates a 50% probability of face and 50% probability of car (50 F). The third indicates a 30% probability of face, 70% probability of car (30 F). After the cue, a blank screen was displayed for a random duration that ranged uniformly between 1.0 and 1.5 seconds. The stimulus image was then presented for 50 ms and participants were given up to 1,250 ms to make their classification response (Supplementary Fig. 4), which was done using a USB button box using their right hand’s index (for face response) and middle (for car response) fingers. No feedback was given about whether the response was correct or incorrect. The trials were presented in 5 blocks of 72 trials, with a 60-second rest period between each block. The entire experiment lasted approximately 25 min. To obtain more statistical power for our single-trial analysis, we asked each participant to perform this task on three consecutive sessions across 3 days which increased the number of trials by a factor of three. Each experiment took place at the same time on each day so that there was 24 h between each session for all participants. For each participant, we made an effort to position the EEG cap in a consistent manner across the three experimental sessions, by keeping the distance between the outermost electrodes and certain anatomical landmarks (i.e., outer canthi, inion, nasion) constant. On the first day, participants performed a practice session of the face/car classification task but with a different set of face and car images. Over the three main experimental sessions, each participant performed a total of 1,080 trials.

### 2.4 EEG data acquisition and preprocessing

Participants performed the task on three experimental sessions, in a dark and soundproof room. During the task, their EEG was recorded with a 64 channel Ag/Agcl scalp electrode actiCAP EEG system (Brain Products GmnH, Gilching, Germany). The active ground electrode was placed just below the Pz electrode of the International 10–20 system. The active reference electrode was placed on the left mastoid. The impedance was always below 5 kOhm for each participant in each session. The EEG signal was acquired at 1,000 Hz with an analogue bandpass of 0.02–250 Hz. The button response and the experimental events codes were also synchronised with the EEG data and collected with the Brain Vision Recorder (BVR; Version 1.10, Brain Products, Germany) software.

We processed the EEG recordings offline using MATLAB. We applied a 0.5-Hz high-pass filter to remove DC drifts and also a 100-Hz low-pass filter to remove high-frequency components not related with neuropsychological processes. These filters were applied together, non-causally to avoid distortions caused by phase delays (using MATLAB “filtfilt”). The EGG data were additionally re-referenced to the averaged for all the 64 EEG channels.

### 2.5 Eye-movement artefact removal

Before the beginning of each experiment, we asked the participants to complete an eye movement calibration task. They were instructed to blink repeatedly upon the appearance of a fixation cross in the centre of the screen and then to make several horizontal and vertical saccades according to the position of the fixation cross on the screen.

The fixation cross subtended 0.4 x 0.4 degree of visual angle. Horizontal saccades subtended 15 degrees and vertical saccades subtended 10 degrees. The timing of these visual cues was recorded with the EEG. We used principal component analysis as described in (L. C. Parra et al., 2005) to determine linear EEG sensor weightings corresponding to 1) eye blinks, 2) horizontal and 3) vertical saccades. These components were then projected onto the broadband EEG data recorded during the main experimental task and subtracted out.

### 2.6 Single-trial discrimination analysis

We performed a linear multivariate single-trial discrimination analysis in order to identify EEG components discriminating the stimulus type (face vs. car) presented on each trial. Here, we closely followed the paradigm established in previous studies (Diaz et al., 2017;Gherman & Philiastides, 2018;Philiastides et al., 2006,^2010^;Philiastides & Sajda, 2006,^2007^), aiming to identify neural activity related to the quality of decision evidence supporting face or car choices. In this approach, the stimulus-locked EEG activity at any time t on any trial*i*, denoted${\vec{x}}_{it}$, is a*K*= 64 column vector. Corresponding to${\vec{x}}_{it}$, we have*z_i_∈ {0,1}*, which is a binary variable that indicates whether the stimulus shown on trial*i*is a face (*z_i_*= 1) or a car (*z_i_*= 0). Our aim is to find a basis vector${\vec{w}}_{t}$that best discriminates the EEG vectors on those trials for which*z_i_*= 0 from the vectors on the trials for which*z_i_*= 1. The value of${\vec{w}}_{t}$can be found by using a logistic regression (L. C. Parra et al., 2005). By finding${\vec{w}}_{t}$that maximises

$$L_{t}=\;\prod_{i=1}^{N}{\text{p}}_{it}^{z_{i}}\;{\left(1-\;p_{it}\right)}^{1-z_{i}}$$ (1)

where

$$p_{it\;}=\;\frac{1}{1+\text{exp}\left(-y_{it}\right)},\;\;\;y_{it}=\;\sum_{k=1}^{K}w_{kt}x_{kit}$$ (2)

and*w_kt_*is element*k*of vector${\vec{w}}_{t}$, and*x_kit_*is element*k*of vector${\vec{x}}_{it}$Note that the (scalar) variable*y_it_*can be seen as a summary representation of the activity in${\vec{x}}_{it}$that best faces (signified by*z_i_*= 1) from cars (signified by*z_i_*= 0). In other words, single-trial amplitudes*y_it_*can be thought of as indexing the quality of the evidence in individual trials, in that a high positive amplitude reflects an easy face trial, an amplitude near zero reflects a difficult trial, and a high negative amplitude reflects an easy car trial.

To apply this discriminant analysis across timepoints, we used a sliding window approach as in previous work. We defined time windows of 50 ms and shifted the window centre in 10 ms increments in a time interval ranging from -100 ms pre-stimulus to 1,000 ms post-stimulus. At each of these timepoints, the EEG activity at each millisecond from 25 ms before to 25 ms after the timepoint was treated as independent observations of the EEG activity at that timepoint. For each of these 50 ms windows, and separately for each participant, we calculate${\vec{w}}_{t}$using logistic regression as explained above.

We performed single-trial classification analyses for each session separately. To maximise the training samples and also to avoid overfitting, we used a leave-one-out cross-validation procedure. Specifically, in each iteration of this cross-validation procedure, N-1 trials are used for training the classifier and the remaining trial is used for testing and this process is repeated N (= No of trials) times. Classification accuracy is the average across the N repetitions. This approach maximises the number of trials used for training the classifier and yields more robust classification estimates. We then quantified the performance of the classifiers using the area under the Receiver Operating Characteristic (ROC) curve, which we label as Az, a widely used method for measuring classification accuracy. The Az value represents the discriminator’s performance over time, with Az = 0.5 reflecting chance performance and Az = 1, reflecting perfect separability between conditions.

The significance of this Az statistic was computed using bootstrapping whereby we randomly permuted classification labels 1,000 times to produce a probability distribution for Az and considered significance at p < 0.01. To assess whether a difference in the reaction times distribution between face and car trials could impact the results of our classification, we also run the discrimination analysis on a subset of trials selected for having matched RT distributions for faces and cars (Supplementary Fig. 1). Specifically, we cut the 8% slowest car trials and 8% fastest face trials for each of the cue three conditions (car-cue, face-cue, neutral-cue). In turn, this resulted in an overall selection of 92% of the original trials. The average RT was now matched between face and car trials in the neutral cue condition, whereas participants were faster on the category corresponding to the cue in the other two (i.e., faster for faces in face-cued trials, and vice versa for car-cued). This manipulation eliminated a potential confound of the difference in RTs.

The discrimination analysis finds the optimal linear combination of EEG activity across all sensors that discriminates faces from cars. Thus, it identifies how much each EEG sensor contributes to achieving maximal discrimination. This contribution is reflected in the scalp topographies, which offer a visual interpretation of the importance/weight of each sensor in achieving this discrimination performance. To compute the scalp projections of the identified discriminating components, we used the forward model formalism:

$${\vec{a}}_{t}=\frac{Xty}{y'y}$$ (3)

where**X***_t_*is the*K× N*matrix formed by concatenating the column vectors${\vec{x}}_{it}$, for all*i*∈ 1 …*N*trials, and**y***_t_*is the*N×*1 vector formed by concatenating*y_it_*for all*i*∈ 1 …*N*trials. This${\vec{a}}_{t}$is known as the*sensor projection*(L. Parra et al., 2003) or*scalp projection*(L. Parra et al., 2002;Philiastides & Sajda, 2006) that can be visualised as*scalp maps*that show the neuroanatomical distribution of the discriminating component. In other words, these forward models can be viewed as scalp topographies and interpreted as the coupling between the observed EEG and the discriminating component amplitudes.

We note that, for the discrimination analysis and all subsequent analyses, we pooled the data together across all sessions and coherence levels in order to increase statistical power. As shown inSupplementary Figure 3, these RT distributions (collapsed across coherence levels) are unimodal. This choice was also consistent with our HDDM formulation in which we did not add a coherence level dependence of the model outputs. When repeating the analysis separately for different coherence levels, we found similar results (Supplementary Fig. 5).

### 2.7 Mixed-effects regression analysis

We used mixed-effects, or multilevel, general and generalised linear models (Gelman & Hill, 2006) for the analysis of the behavioural and EEG data. Specifically, we used a general linear model (lineal regression) to analyse RTs and generalised linear models (logistic regression) for accuracy data. These models allow us to model inter-participant variability and to combine continuous and categorical variables in the analysis of outcome variables, which themselves may be continuous or categorical. In these models, the inclusion of the random effects term accounts for the inter-participant variability around population level average effects, thereby avoiding inflated Type I error rates (Aarts et al., 2014).

The significance of a single variable, or set of variables, in the multilevel regression models is tested using a log likelihood ratio test. To test the significance of a size*K*^′^subset of all*K*predictor variables, we compare the log-likelihood of the model with all*K*predictors against the log likelihood of the model without the*K*^′^subset. If we denote the log likelihood of the model with all K predictors by*L*_1_and the log likelihood of the model with the subset*K*^′^by*L*_0_, then under the null hypothesis that all coefficients corresponding to the*K*^′^predictors are simultaneously zero, −2 × (*L*_0_−*L*_1_) ~$\chi_{df}^{2}$where the degrees of freedom of the χ^2^statistic is the difference in the number of predictors between the two models.

Here, we fit all multilevel linear and logistic regression models using the lme4 package (Bates et al., 2015) in the R statistical computing language (R Core Team, 2021).

### 2.8 Neurally-informed hierarchical drift diffusion model

We used a hierarchical drift diffusion model (HDDM) (Ratcliff, 1978;Ratcliff & McKoon, 2008) to model participants’ perceptual choices and reaction times (RTs). This model assumes the random diffusion of a decision variable that represents the accumulation of evidence for one or the other of the two alternative choices, that is, face or car here. The choice that is made in a decision task and the response time taken to make this choice are modelled by the probability and time of crossing the upper or lower boundary. The HDDM estimates parameters representing internal components of processing such as the rate of evidence accumulation (drift rate), the distance between decision boundaries controlling the amount of evidence required for a decision (decision boundary), a possible baseline bias towards one of the two choices (starting point) and the duration of non-decision processes (non-decision time), which include stimulus encoding and response production. The hierarchical implementation of this model includes random effects on the model parameters to model variability across participants or across conditions in the effect of the predictors on the observed data (Vandekerckhove et al., 2011). Unlike traditional DDM requiring variance in drift rate, in the Bayesian framework this variability can be captured by the uncertainty in the parameter estimations. HDDM estimates all parameters as random variables (RVs) with probability distribution functions (means and variances), thus the drift rate (and the other parameters) varies a) from trial-to-trial and b) from participant-to-participant.

Thus, in this hierarchical formulation, the HDDM parameters were obtained using Bayesian inference, whereby expected values of the model parameters were updated on the basis of the likelihood of the data under the model and their prior distributions (Kruschke, 2010;Wabersich & Vandekerckhove, 2014;Wiecki et al., 2013). The use of Bayesian models, and specifically the Bayesian HDDM diffusion model, has several benefits relative to traditional DDM analyses. First, this framework supports the use of other variables as regressors of the model parameters to assess relations of the model parameters with physiological or behavioural signals of interest (Cavanagh et al., 2014;Delis et al., 2018;Frank et al., 2015;Nunez et al., 2015;Pedersen et al., 2017;Turner et al., 2015). This property of the HDDM enabled us to inform the model with EEG signatures of the neural evidence available for perceptual choice. Second, the model estimates posterior distributions of the main parameters (instead of deterministic values), which directly convey the uncertainty associated with parameter estimates (Gelman, 2003;Kruschke, 2010). Third, the Bayesian hierarchical framework has been shown to be especially effective when the number of observations is low (Ratcliff & Childers, 2015).

We used the JAGS Wiener module (Wabersich & Vandekerckhove, 2014) in JAGS (Plummer, 2003), via the Matjags interface in MATLAB to implement the HDDM here. Parameters were drawn from uniformly distributed priors and were estimated with non-informative mean and standard deviation group priors. There were 11,000 samples drawn from the posterior. The first 1,000 (burn-in) samples were discarded, as initial samples are likely to be unreliable due to the selection of a random starting point. The rest of the samples were subsampled (“thinned”) by a factor of 50 as neighbouring samples are likely to be highly correlated (Wabersich & Vandekerckhove, 2014;Wiecki et al., 2013). The remaining samples constituted the probability distributions of each estimated parameter. To ensure convergence of the model, we used the Gelman-Rubin$\hat{R}$statistic and verified that all group-level parameters had an$\hat{R}$close to 1 and always lower than 1.03.

Our primary aim here was to determine how prior probability of the upcoming stimulus, as revealed by the pre-stimulus cue, affects perceptual decision making by examining which parameters of the diffusion model, and consequently which underlying processes, are dependent on the stimulus prior probability. For example, evidence for the prior probability biasing the baseline of the available evidence would be obtained by observing changes in the starting point variable in the HDDM. On the other hand, evidence of prior probability affecting the quality of the available evidence would be obtained by observing changes in the drift rate. To arbitrate between these alternatives, we estimated different neurally-informed HDDMs in which EEG activity representing the relevant decision evidence on individual trials was used to better explain the trial-wise variability in the drift rate and/or starting point parameters in the model. Specifically, we used the single-trial amplitudes (*y*values) of two identified EEG components (Early and Late) discriminating between face and car trials as regressors of the two HDDM parameters of interest (drift rate and starting point).

Therefore, as part of the model fitting of the behavioural data (single-trial choices and RTs) within the HDDM framework, we used the*y*values of the EEG components as regressors of the single-trial drift rate δ*^i^*(or starting point β*^i^*) as follows:

$$\delta i=\gamma_{0}+\gamma_{Early}\cdot y_{i}^{Early}+\left(\gamma_{Late}\cdot y_{i}^{Late}\right)\cdot C_{i},$$ (4)

where$y_{i}^{Early}$and$y_{i}^{Late}$are the single-trial discriminator amplitudes of participant-specific stimulus-locked*Early*EEG components (defined as individual peak Az in the time range 150–250 ms post-stimulus) and Late EEG components (individual peak Az in the time range 300–500 ms post-stimulus), respectively. The coefficients γ_Early_and γ_Late_weight the slope of the drift rate by the values of$y_{i}^{Early}$and$y_{i}^{Late}$on that specific trial*i*, with an intercept γ0.

The variable*Ci*is the coherence level of the image presented on each trial. This value represents the quality of visual evidence available on each trial and has been shown to be proportional to the amplitude of the Late component (Philiastides et al., 2006;Philiastides & Sajda, 2007;Ratcliff et al., 2009). Hence, by using these regression coefficients, we were able to test the influences of each of the two identified components on the drift rate (or starting point) for the three prior probability cues. Overall, we tested five different neurally-informed HDDMs (nHDDMs): 1) with$y_{i}^{Early}$and$y_{i}^{Late}$as regressors for starting point β, that is, both components as predictors of a bias in the baseline of stimulus evidence, 2) with$y_{i}^{Early}$as regressor for starting point β and$y_{i}^{Late}$as regressor for drift rate δ, that is, the Early component as predictor of a bias in the baseline and the Late component as predictor of the quality of decision evidence, 3) with$y_{i}^{Early}$and$y_{i}^{Late}$as regressors for drift rate δ, that is, both components as predictors of the quality of stimulus evidence entering the decision, 4) with only$y_{i}^{Early}$as regressor for drift rate δ, and 5) with only$y_{i}^{Late}$as regressor for drift rate δ.

For comparison, we also fit a behaviour-only HDDM (without EEG regressors) to the behavioural data. In this model, drift rate, boundary separation, starting point and non-decision time were estimated for each individual participant and were dependent on the presented cue.

To compare between the above candidate models, we employed the Deviance Information Criterion (DIC), a measure widely used for fit assessment of hierarchical models (Spiegelhalter et al., 2002). DIC selects the model that achieves the best trade-off between goodness-of-fit and model complexity (lower values are better). After choosing the best model, posterior probability densities of each regression coefficient were estimated using the Monte Carlo sampling procedure described above. Positive (negative) effects were determined when >95% of the posterior density was higher (lower) than 0. All statistical tests at the population level were performed by contrasting the population-level distributions (not the individual participant means) across stimulus probabilities.

This statistical testing takes into account the hierarchical structure of the model and has been shown to reduce biases and actually yield conservative effect sizes (Boehm et al., 2018).

## 3 Results

**Fig. 1.**
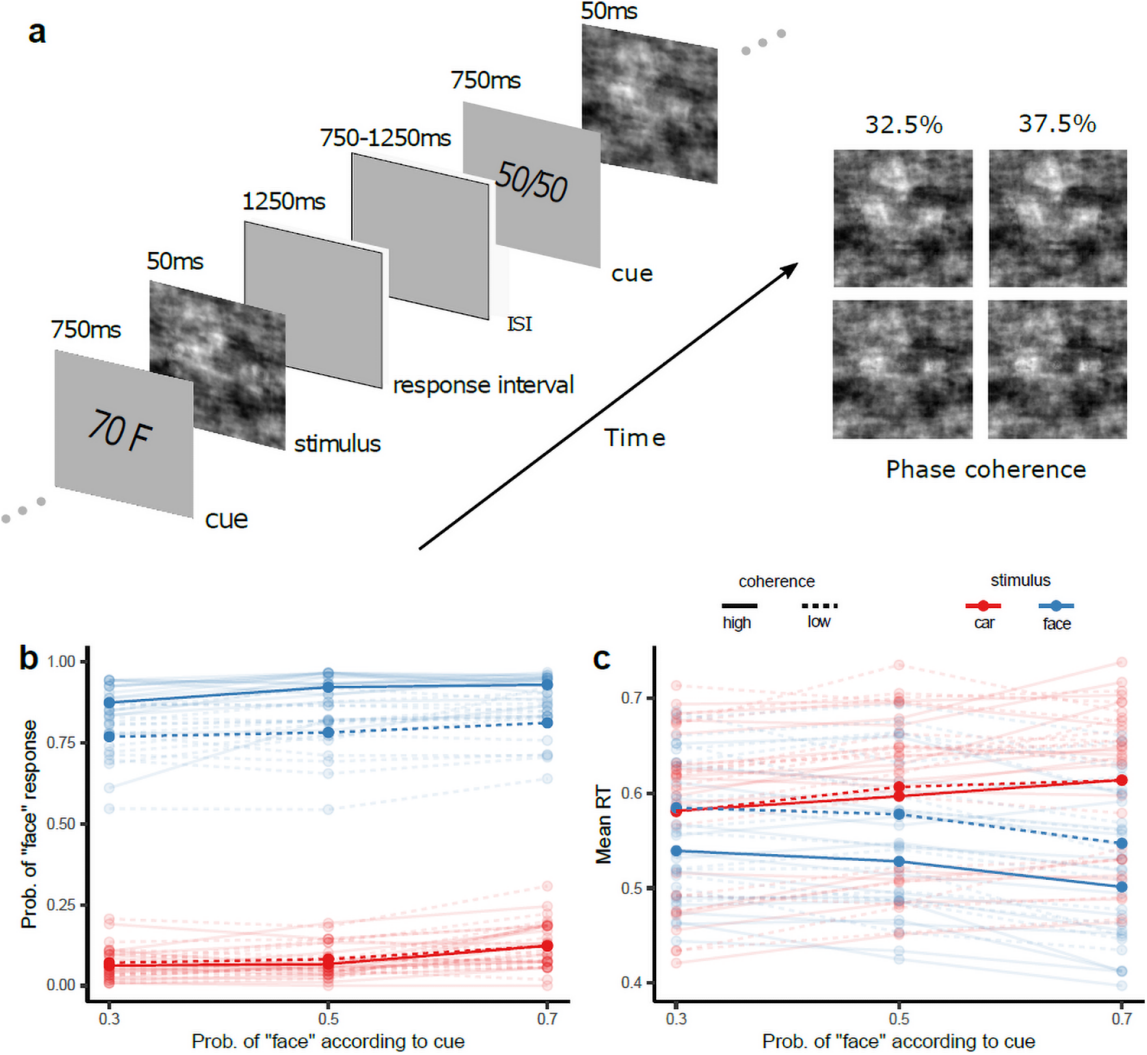
Experimental design and behavioural analysis. (a) Schematic representation of the experimental paradigm. During the EEG experiments, participants had to categorise a noisy image presented for 50 ms as either a face or a car and indicate their choice with a button press within 1,250 ms following the stimulus presentation. Prior to stimulus onset, a cue was shown indicating the probability of the subsequent stimulus being either a face or car, followed by an inter-stimulus interval that varied randomly between 1 and 1.5 s. Participants performed this task on three different sessions (i.e., on three consecutive days). Examples of face (top) and car (bottom) images at two different % phase coherence levels are shown on the right. (b) Probability of a face response and (c) average reaction time as a function of stimulus probability for the two stimulus types (face: blue, car: red). Faint lines are individual participant responses. Solid and dashed lines represent high and low image coherence levels, respectively.

We presented 16 human participants in three different sessions with noisy images of faces and cars and instructed them to decide as quickly and as accurately as possible whether each stimulus contained one or the other image category (face or car) while we collected behavioural (choice-RT) and EEG data. To manipulate task difficulty, we adjusted the percentage of phase coherence of the images (two levels; 32.5% and 37.5%). Crucially, prior to the presentation of the stimulus (face or car), we presented a cue that informed participants of the probability that the upcoming image would contain a face (three levels: 30%, 50%, or 70% face probability; stimulus cues: 30 F, 50 F, 70 F) (Fig. 1aandSupplementary Fig. 4), thus manipulating the prior expectations of the participants.

### 3.1 Prior probability effects on perceptual choice

We used a mixed-effects binary logistic regression to model the effect of stimulus type (face or car), pre-stimulus cue probability, and their interaction, on the probability of face choice. To obtain higher statistical power, we pooled our behavioural data across the three experimental sessions and collapsed across the two-phase coherence (i.e., difficulty) levels. We found a significant effect of stimulus type ($\chi_{1}^{2}$= 11,357.17,*p*≪ 0.01), a significant effect of cue probability ($\chi_{1}^{2}$= 82.47,*p*≪ 0.01), and no interaction ($\chi_{1}^{2}$= 2.16,*p*= 0.14). As expected, there were more face choices for face stimulus trials than car stimulus trials (and vice versa). More importantly, as the cue indicated a higher probability of face, the probability of face choice increased for both face and car stimuli trials and for both phase coherence levels (Fig. 1b).

To assess reaction time (RT) effects, we used a mixed-effects linear regression where we modelled the logarithm of reaction time as a function of the stimulus type, the probability of the upcoming stimulus according to the cue, and their interaction. There was a significant effect of stimulus type ($\chi_{1}^{2}=735.46,p\;\ll 0.01$), a significant effect of stimulus probability ($\chi_{1}^{2}=$242.93,*p ≪*0.01), and an interaction between the two ($\chi_{1}^{2}=5.05,p=0.02$). Specifically, face choices were generally faster on average than car choices (the difference in the average reaction time to face and car stimulus was 57 ms, with a standard deviation of 42 ms). More importantly, as the probability of face increased according to the cue, reaction times decreased on face trials and increased on car trials (for both coherence levels,Fig. 1c). Overall, we found that prior probability biases perceptual choice by increasing the number of choices of the most likely stimulus (accord- ing to the cue) and decreasing (increasing) RTs when the stimulus is congruent (incongruent) with the cue probability.

### 3.2 A mechanistic account of prior probability effects

Traditional (behaviour-only) modelling studies have thus far offered contradicting views on whether prior probability effects are driven by changes in the starting point or the drift rate of evidence accumulation. Here, to disambiguate between these different (but equally likely) computational accounts of choice-RT data, we aimed to integrate EEG activity in the estimation of a hierarchical drift diffusion model (HDDM) (Vandekerckhove et al., 2011), thus forming neurally-informed HDDMs (nHDDM). We focused on two temporally distinct neural components that are known to reflect the early sensory as well as the post-sensory evidence entering the decision process respectively (Blank et al., 2013;Delis et al., 2016;Lou et al., 2014;Philiastides et al., 2011;Philiastides & Sajda, 2006,^2007^), thus offering a path to better accounting for the internal variability in the encoding of the evidence in the nHDDM model. Specifically, these two EEG components discriminate between the stimulus categories (faces-vs-cars): an Early component, appearing 200 ms post-stimulus onset, and a Late component, seen after 300–500 ms following the stimulus presentation. Previous work has found that both of these components are predictive of behaviour but with the Late component being a better predictor of choice accuracy, as it predicted changes in the rate of evidence accumulation in a traditional DDM and shifted later in time with longer deliberation times (Diaz et al., 2017;Franzen et al., 2020;Philiastides et al., 2006;Ratcliff et al., 2009).

**Fig. 2.**
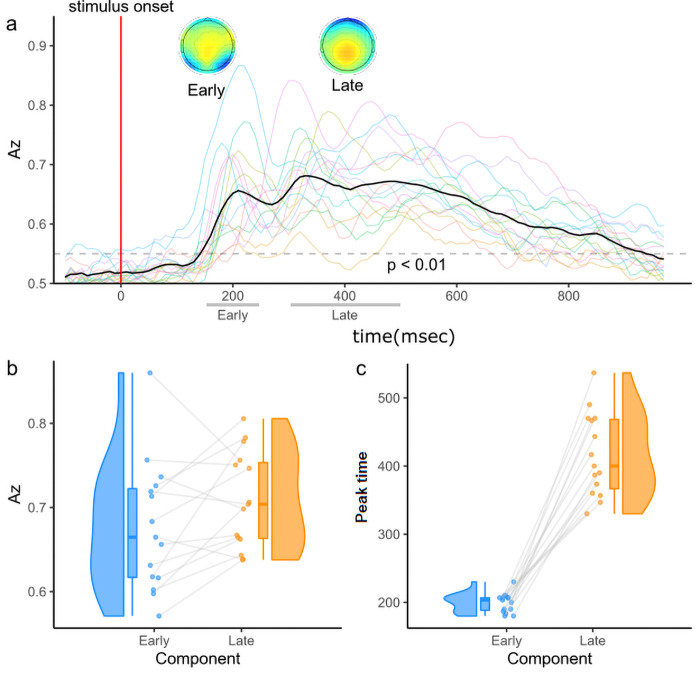
Single-trial EEG components. (a) Multivariate single-trial discriminator performance (*A_z_*) during face versus car discrimination on stimulus-locked EEG data, averaged across participants and sessions, showing the presence of an Early and Late component. Faint lines represent individual participant data. In the insets, the topography of the two components, representing which electrodes carry more weight for the discriminating component, that is, more discriminating power. The plots represent population averages of the forward model (Equation 3) of each participant at peak time. Boxplots with individual data along with a density plot showing (b) the average discriminator performance for the Early (light blue) and Late (orange) components and (c) the average peak times for the Early and Late components, estimated at the time of participant-specific peak discrimination. Data points corresponding to the same participant at the Early and Late components are joined by a line.

To this end, we deployed a face versus car discrimination analysis on our stimulus-locked EEG signals to identify the neural responses related to the quality of decision evidence supporting face or car choices. The discrimination analysis finds the optimal linear combination of EEG activity across all sensors that best discriminates between faces from cars. We identified two EEG components discriminating between face and car trials (Fig. 2aandSupplementary Fig. 1) consistent with the work outlined above. Specifically, we identified an Early component with an average peak at approximately 200 ms distributed across occipito-parietal sensors and a Late component with an average peak at approximately 350 ms characterised by a centroparietal positivity typically found in many perceptual decision tasks (Kelly & O’Connell, 2015).

To analyse the relative strength of each component (*A_z_*) as well as their relative latencies, we used a multilevel (mixed-effects) linear model, modelling Az value as a function of component, with a random intercept for participants to account for inter-participant variability. As with the behavioural analysis, to achieve higher statistical power, we pooled the Az values across all three experimental sessions and coherence (i.e., difficulty) levels (results were similar when analysing trials with different coherence levels separately—Supplementary Fig. 5). We found a highly significant effect of component on both*A_z_*(χ^2^= 9.83,*p*< 0.01;Fig. 2b) and onset time (χ^2^= 159.05,*p*< 0.01;Fig. 2c), with the Late component reaching higher discrimination power and occurring systematically later in time compared to the Early one.

**Fig. 3.**
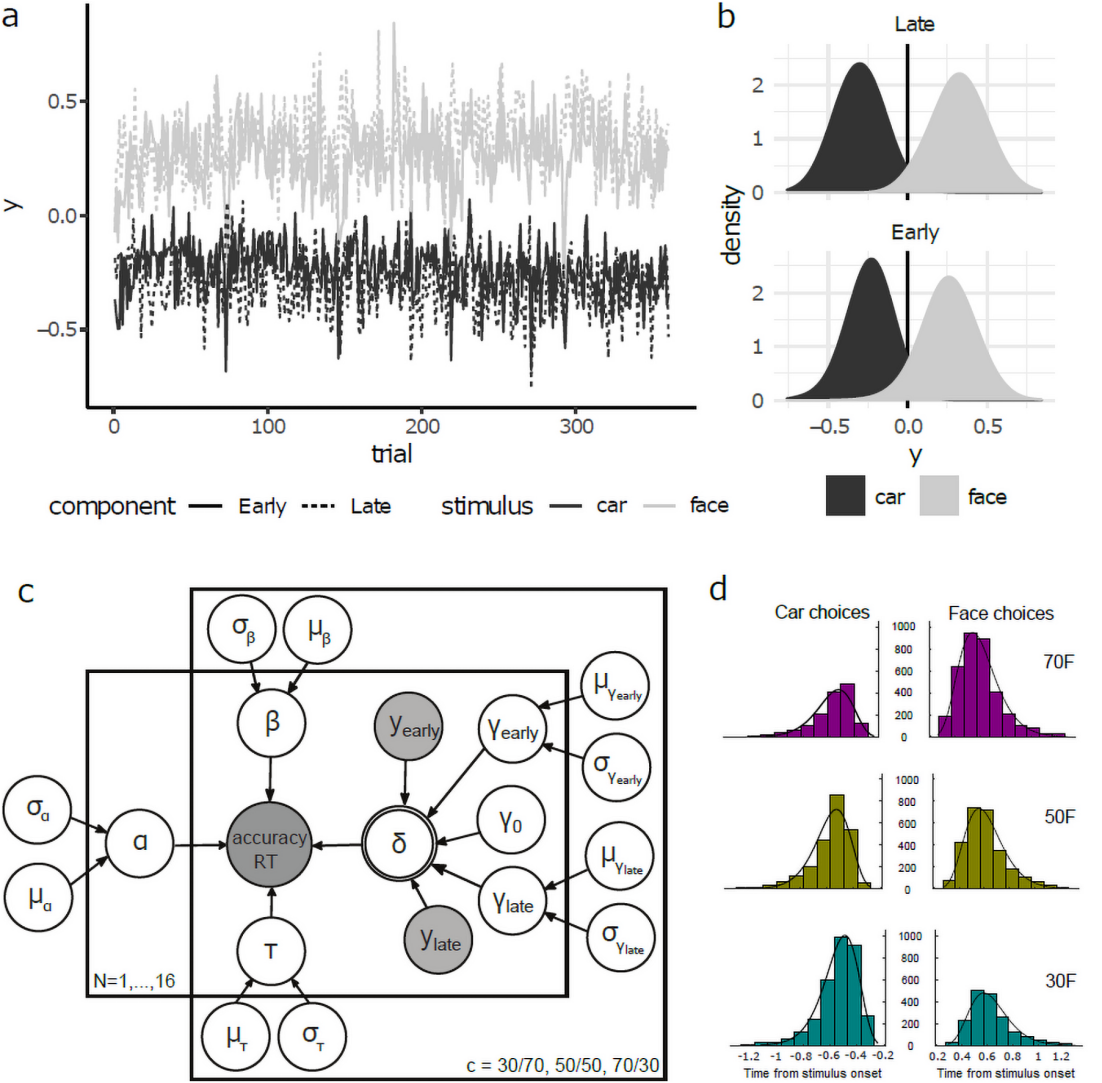
Neurally-informed HDDM. (a) Single-trial discriminator amplitudes (*y*) for the Early (solid lines) and Late (dashed lines) components for face (light grey) and car (dark grey), averaged over all participants and training days. (b) The same single-trial discriminator amplitudes shown as density plots (using Gaussian kernel density estimator with a standard deviation of 0.1). Also shown is the threshold of*y*= 0. When*y*> 0, this corresponds to a higher probability of a face stimulus, and*y*< 0 corresponds to a higher probability of a car stimulus. (c) Graphical representation showing hierarchical estimation of HDDM parameters. Round nodes represent continuous random variables and double-bordered nodes represent variables defined in terms of other variables. Shaded nodes represent recorded or computed signals, that is, single-trial behavioural data (choice, RT) and EEG component amplitudes (*y*’s). Parameters α (decision boundary), β (starting point), τ (non-decision time), δ (drift rate) as well as the intercept and two regression coefficients γ_0_, γ*_Early_*, γ*_Late_*of the drift rate linear regression model are modelled as random variables with inferred means µ and variances σ^2^. Plates denote that multiple random variables share the same parents and children (one over participants and another one over prior probability cues). (d) Histogram and model fits (solid line for nHDDM and dashed line for behavioural HDDM) for RT distributions of car (left) and face (right) choices for the three levels of prior probability.

In this analysis, single-trial amplitudes (*y*) from the two EEG components can be thought of as indexing the quality of the evidence in individual trials, in that a high positive amplitude reflects more face evidence, an amplitude near zero reflects a more ambiguous trial, and a high negative amplitude reflects more car evidence (Fig. 3a, b). To quantify the relative contribution of each component to the eventual choice, we used the single-trial amplitudes of the two components (*y*’s;Fig. 3a) as predictors of face choice in a logistic regression. The Early and Late*y*’s were normalised (scaled to have a standard deviation of 1.0), to enable direct comparisons of the regression coefficients from each predictor. We found that both the Early and Late component amplitudes predicted the probability of a face response (95% confidence intervals are [0.88, 0.985] and [1.065, 1.174] for the regression coefficients, and 95% confidence intervals for the odds ratios are [2.41, 2.679] and [2.902, 3.234], of the Early and Late*y*’s respectively), but the Late component had significantly higher predictive power ($\chi_{\left[1\right]}^{2}$= 20.43,*p*< 0.001). These results are consistent with a long body of previous work indicating that the amplitudes of the Early and Late EEG components are reliable indices of the quality of the neural evidence entering the decision, with the Late component in particular being an overall better predictor of the eventual choice (Philiastides et al., 2006;Philiastides & Sajda, 2006,^2007^;Ratcliff et al., 2009).

Having identified these neural signatures of face/car decision evidence, we then asked how trial-wise fluctuations in these internal representations might help explain additional variance in the estimation of choice-RT data in a nHDDM (and thereby offer a more accurate mechanistic account of the role of prior probability on choice behaviour). HDDMs decompose decision-making performance (i.e., choice and RT) into internal components of processing representing the rate of evidence integration (drift rate, δ), a possible prior bias toward one or the other choice (starting point of the evidence accumulation, β), the amount of evidence required to make a choice (boundary separation, α), and the duration of other processes, such as stimulus encoding and response production (non-decision time, τ). Crucial to our investigation, parameters (β and δ) represent the potential “baseline” bias towards one of the two choices and changes in the quality of evidence used in the decision due to the prior probability manipulation, respectively. Thus, by comparing the obtained values for these parameters across stimulus prior probabilities, we could associate any behavioural differences to the constituent internal process instantiated by each HDDM parameter.

We aimed to understand if the use of a neurally-informed HDDM provides any benefit compared to traditional (behaviour-only) alternatives. Thus, we first tested a standard HDDM that did not include any neural correlates. Consistent with results of standard sequential sampling models (Forstmann et al., 2010;Leite & Ratcliff, 2011;Mulder et al., 2012), the behavioural HDDM yielded differences in the starting point across stimulus probabilities (Prob (β70F > β30F)> 0.97 and Prob(β50F > β30F)> 0.90) but also strong differences in drift rates (Prob (δ70F > δ50F)> 0.999 and Prob(δ50F > δ30F)> 0.999).

We then informed the nHDDM with the single-trial discriminator amplitudes of the Early and Late EEG components identified above ($y_{i}^{Early}$and$y_{i}^{Late}$respectively) and treated them as indices of the amount of neural evidence available for a face or car choice (i.e., more positive values indicating face evidence and more negative values indicating car evidence,Fig. 3a, b). While the amplitudes of the two EEG components have previously been shown to correlate with the drift rate of traditional DDMs (Philiastides et al., 2014;Ratcliff et al., 2009), in principle they could still reflect the amount of baseline evidence entering the decision prior to evidence integration (i.e., the starting point in the DDM), when prior probability is manipulated explicitly. To assess potential contributions of the two components to the baseline and/or the accumulation of decision evidence from prior probability, we employed$y_{i}^{Early}$and$y_{i}^{Late}$as regressors in the following three nHDDMs: 1) including the two components as regressors of starting point, 2) having y_Early_as regressor for starting point and y_Late_as regressor for drift rate, and 3) having both components as regressors for drift rate.

We found that model 3 provided a remarkably better fit than the other two regression models (achieving a better complexity-approximation trade-off, DIC_1_= 815, DIC_2_= -1,733 and DIC_3_= -3,252, seeFig. 3cfor a schematic illustration of nHDDM 3 andFig. 3dits fits of choice-RT data), indicating that the inclusion of both EEG components as predictors of drift rate on a trial-by-trial basis led to a better approximation of the participants’ single-trial behavioural data. Interestingly, models 1 and 2, besides offering poorer data fits, also showed no effect of prior probability on starting point regression coefficients. Crucially, nHDDM model 3 also yield a better fit to the choice-RT data than the behavioural alone HDDM (DICnHDDM = -3,292 vs. DICHDDM = -1,552). Specifically, the main reason why the behavioural HDDM underperformed compared to the neurally-informed HDDM was its tendency to underestimate the number of trials with longer RTs and slightly overestimate those with shorter RTs. Given the poorer fit of the choice and reaction time data, this finding suggests that constraining the HDDM with neural measures can help disambiguate between competing hypotheses about the behavioural effects observed.

To understand how the inclusion of the neural measures improves model fitting, we first evaluated the contribution of the two components to trial-to-trial drift rate variations. We found that the linear model of drift rate that includes both EEG components as regressors is a good fit of the estimated drift rate (R^2^= 0.91) and that the two EEG components contribute highly to this approximation (R^2^of the model including the two regressors and no constant term is 0.71), thus suggesting that the EEG components enable a better approximation of drift rate modulations in single trials, which may lead to a better account of the single-trial decision dynamics, compared to the traditional HDDM that contains no neural regressor for drift rate.

To further validate this observation in light of alternative model formulations, we tested variants of the best neurally-informed model 3, which included only one of the two component amplitudes (i.e., y_Early_or y_Late_) as drift rate regressor. We found that model fits were poorer than for the two-component model (DIC = -3,252 for the original nHHDM versus -2,273 and -2,661 for the models using only the Early or only the Late component respectively), thus the two-component model provided the best approximation of the behavioural measurements by accounting for single-trial variations of the drift rate.

Taken together, these results indicate that the two components did not associate with prior probability modulations of the baseline of decision evidence. We thus used nHDDM 3 (with both components as regressors for drift rate) to further investigate the mechanistic effect of prior probability on perceptual choice.

**Fig. 4.**
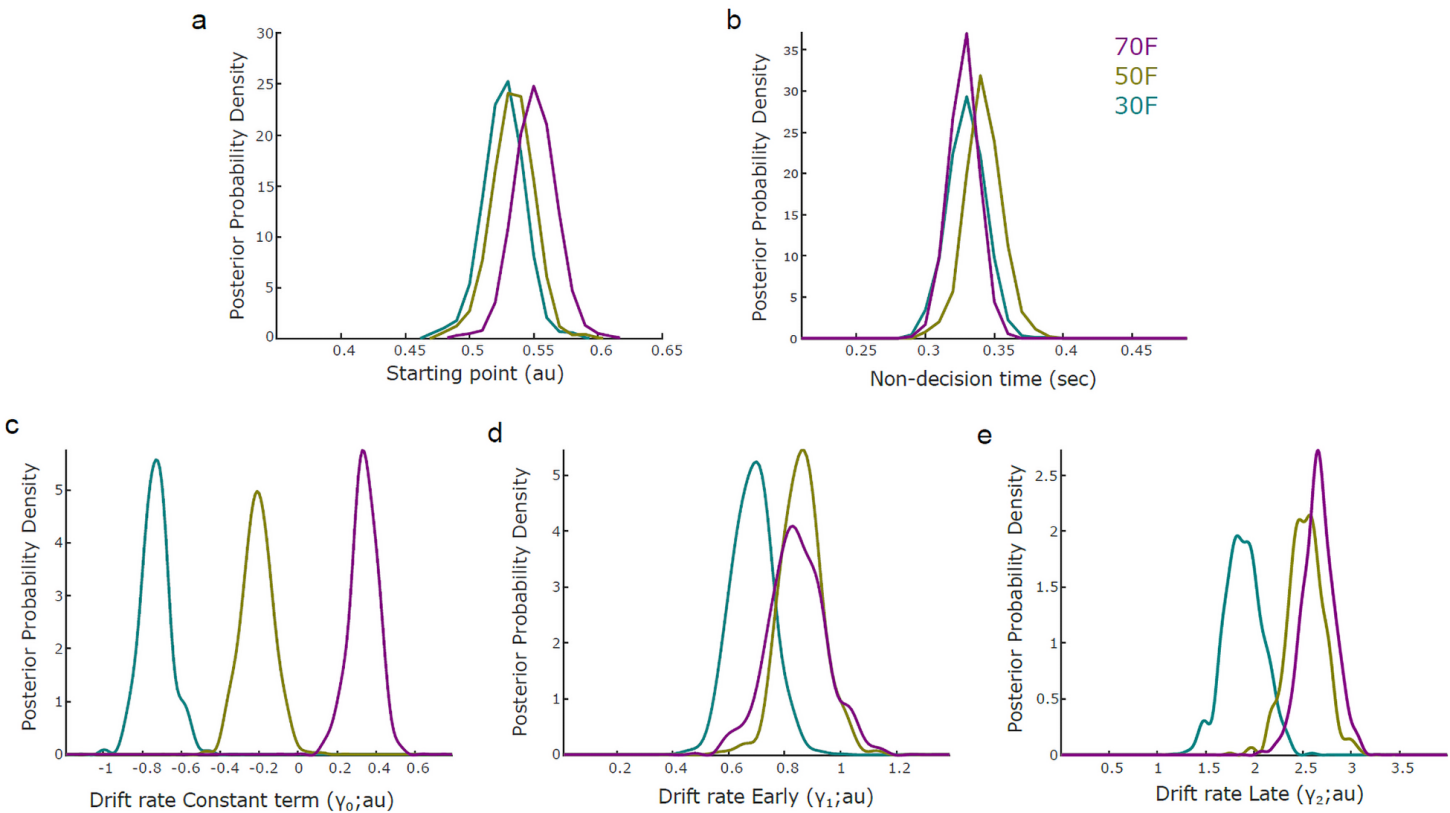
nHDDM output parameters (a, b). Posterior probability distributions of starting point β (in a) and non- decision time τ (in b) estimated by the nHDDM for the 70% probability of face (70 F; pink), 50% probability of face (50 F; yellow) and 30% probability of face (30 F; blue) stimulus probability cues. (c, d, e). Posterior probability distributions of regression coefficients (γ_0_in c, γEarly in d, γLate in e) as predictors of the drift rate (δ) of the nHDDM. Γ_0_represents the constant term of the regression and γEarly, γLate are coefficients of the Early (middle) and Late (right) EEG component amplitudes (*y*’s) respectively. Coefficients were derived from the nHDDM including n = 16 independent participants and 17,280 trials.

Specifically, we tested whether the two component amplitudes ($y_{Early}^{s}$and$y_{Late}^{s}$) were predictive of drift rate in single trials across participants and whether there was a differential effect of prior probability on these modulations. We found strong positive modulations of drift rate from both components (*Prob*γ*_Early_*> 0 > 0.999 and*Prob*(γ*_Late_*> 0) > 0.999 for all probability cues;Fig. 4d, e), further validating the role of these components in representing the quality of evidence available for a choice. Note that a nHDDM in which only the Late component amplitude scaled with stimulus phase coherence fit the data better than an alternative nHDDM where both components scaled with phase coherence (DIC = -3,292 vs. -3,225), which is consistent with the behavioural results indicating that the Late component is ultimately more closely associated with task demands and the eventual accuracy.

Crucially for our main investigation of the effects of prior probabilities, we also found that the relationship between drift rate and component amplitudes was modulated by the stimulus prior probability. Specifically, we found stronger differences in γ*_Late_$(Prob(\gamma_{Late}^{70F}>\;\gamma_{Late}^{30C}$*) > 0.99 and$(Prob(\gamma_{Late}^{50F}>\;\gamma_{Late}^{30F}$) > 0.99;Fig. 4e) and slightly weaker differences in γ*_Early_$(Prob(\gamma_{Early}^{70F}>\;\gamma_{Early}^{30F}$*) > 0.87 and*$(Prob(\gamma_{Early}^{50F}>\;\gamma_{Early}^{30F}$*) > 0.93;Fig. 4d) across the three levels of prior probability. Taken together, these findings suggest a strong influence of prior probability on the quality of decision evidence.

Finally, we investigated the effect of prior probability on the other nHDDM parameters of winning model 3. Importantly, we found no reliable relationship between prior probability and starting point (*Prob*(β^70F^> β^30F^) = 0.85,*Prob*(β^50F^> β^30F^) = 0.63,*Prob*(β^70F^> β^50F^) = 0.79;Fig. 4a), indicating that prior probability was unlikely to induce a reliable perceptual choice bias via modulations of the baseline of decision evidence. Interestingly, the higher number of face trials (70%) following the 70 F cue and car trials (70%) following the 30 F cue was not reflected in starting point differences, that is, a starting value of 0.3 for the 30 F cue, 50 for the 50 F cue and 0.7 for the 70 F cue as would be expected based on the respective stimulus probabilities. There was also no influence of prior probability on non-decision time (Prob (τ70F > τ30F) = 0.46, Prob (τ50F > τ30F) = 0.75, Prob (τ70F > τ50F) = 0.20;Fig. 4b), indicating no effect of prior probability on the sensory processing or motor response duration.

Instead, our nHDDM analysis revealed a significant modulation of the intercept term γ_0_of the drift rate regression from the stimulus prior probability (Fig. 4c). This is reflected in a positive$\gamma_{0}$for the 70 F cue (*Prob($\gamma_{0}^{30F}$*> 0) > 0.99) and a negative$\gamma_{0}$for the 30 F cue (*Prob($\gamma_{0}^{30F}$*< 0) > 0.999) and the 50 F cue (*Prob*$\gamma_{0}^{50F}$< 0 > 0.99). Thus, the different stimulus probabilities were captured by modulations of the intercept term of drift rate (positive for more face trials and negative for more car trials). This also captures the slight bias towards car choices (negative γ_0_) in the unbiased stimulus probability condition 50 F (which can also be observed in the histograms ofFig. 3d). Taken together with the above, these results indicate that the choice biases induced by prior expectation and stimulus probability are explained by drift rate, rather than starting point, modulations.

Overall, the above findings suggest that the two EEG components are reliable predictors of drift rate, rather than starting point, modulations in general and of the effect of prior probability on such modulations in particular. Inclusion of these components in the HDDM not only improved the approximation of the behavioural data but also helped disambiguate the mechanistic effect of prior probability on choice behaviour.

## 4 Discussion

In this study, we used computational modelling coupled with multivariate decoding of EEG signals to probe the mechanistic influence of prior probability on the processes underpinning perceptual decision formation. We introduced a neurally-informed modelling approach that enabled us to dissect this effect and showed that prior probability biases primarily the accumulation of the evidence in the decision process, rather than the baseline activity entering the decision process. Our behavioural results demonstrated that the prior probability of an upcoming stimulus (presented as pre-stimulus cues) biased perceptual choices. As the probability of a particular stimulus increased, so did the probability of choosing that stimulus. Similarly, if the stimulus was congruent (incongruent) with the prior probability cue, the speed of the choice increased (decreased). These behavioural findings are in line with already established results in the literature (see, e.g.,Aslin et al., 1998;Auckland et al., 2007;Bar, 2004;Davenport & Potter, 2004;Fiser & Aslin, 2002;Oliva & Torralba, 2007;Palmer, 1975;Saffran et al., 1996).

When investigating the neural mechanisms underpinning these behavioural effects, we first identified EEG signatures of the evidence entering the decision process. Specifically, our single-trial EEG analysis yielded two components reflecting face versus car neural evidence: an Early one occurring at approximately 200 ms, and a Late one occurring around 350 ms, with the Late one having higher predictive power in explaining the upcoming choice. These EEG components are fully consistent with prior work and served as reliable neural signatures of stimulus evidence in single trials (e.g.,Delis et al., 2016;Diaz et al., 2017;Philiastides et al., 2006,^2010^;Philiastides & Sajda, 2006,^2007^). These neural signatures were used to inform a computational model of decision-making behaviour, namely a HDDM, in order to understand the mechanistic influence of prior probability on perceptual choice. Although sequential sampling models, in general, and drift diffusion models, in particular, have been remarkably successful at modelling behavioural data (e.g.,Arabadzhiyska et al., 2022;Bolam et al., 2024;Evans & Wagenmakers, 2020;Gabay et al., 2024;Pisauro et al., 2017;Ratcliff & McKoon, 2008;Ratcliff & Smith, 2004;Ratcliff & Van Dongen, 2011;Smith & Ratcliff, 2004;Usher & McClelland, 2001), and can in principle exploit differences in RT distributions in order to provide evidence for different hypotheses (Balsdon et al., 2023;Mulder et al., 2012;Verdonck et al., 2021), it is not always possible to differentiate between models with different underlying assumptions that under certain conditions make similar predictions about behavioural data (Kelly et al., 2021).

Thus, supplementing behavioural measurements with the underlying neural signals can increase the explanatory power of these models and also offer a better mechanistic understanding of how cognitive processes are implemented at the level of neural responses (Delis et al., 2018;Frank et al., 2015;Franzen et al., 2020;O’Connell & Kelly, 2021;Turner et al., 2015).

Here, we employed such an approach to obtain a mechanistic understanding of how prior probability biases perceptual choices in a simple visual categorisation task. To date, two main accounts have been proposed to explain the effects of prior probability. While the conventional account suggests a bias in the starting point of evidence accumulation, more recent reports propose an additional bias on the accumulation of the available evidence (i.e., drift rate bias) (Summerfield & Egner, 2009). Support for the former account comes from changes in baseline activity in middle temporal area neurons coding for the effect of experience on relevant sensory evidence (i.e., motion discrimination) in non-human primates (Albright, 2012) as well as expectation-driven baseline changes in inferior temporal cortex in humans during a category-informed face-house discrimination task (A. M. Puri et al., 2009). More recently, further support for such a baseline offset of the perceptual evidence for decision-making was found in prefrontal cortex neurons of macaques during an orientation discrimination task (Charlton & Goris, 2024). Similarly, this account is consistent with the predictive coding hypothesis (Friston, 2005;Rao & Ballard, 1999) which posits that predictions based on prior information are compared to actual sensory input to bias activity in early sensory cortex (Kok et al., 2012,^2017^).

Recent studies have also employed computational modelling to help elucidate the mechanistic origins of such decision-making biases (Cerracchio et al., 2023;Huang et al., 2012). Crucially, novel findings from cognitive models of decision-making have started to challenge the selective influence of prior probability on a baseline bias, indicating that it can also affect evidence encoding (Walsh et al., 2024) accumulation (Cerracchio et al., 2023) or non-decision processes (R. Puri et al., 2023).

Here, by fitting a behaviour-only HDDM on our data, we identified an effect of prior probability on both the starting point and drift rate, consistent with both the accounts (K. Dunovan & Wheeler, 2018;K. E. Dunovan et al., 2014;Kelly et al., 2021). The behaviour-only HDDM therefore did not allow us to disambiguate with confidence between the two different hypotheses. Surprisingly, however, we found no evidence of the effect of prior probability on the starting point when deploying our neurally-informed HDDM, which instead suggests that the observed prior probability effects are driven primarily by the accumulation of decision evidence (i.e., changes on drift rate alone). This finding corroborates evidence from non-human primate work reporting increases in the firing rate of evidence accumulation regions in parietal cortex (i.e., lateral intraparietal area) as a result of prior probability manipulations (Hanks et al., 2011) and in humans where prior expectations have been associated with changes in the rate of evidence accumulation in the DDM (Cravo et al., 2013) as well as changes in neural activity in dorsolateral prefrontal cortex and its effective connectivity with sensory regions (Rahnev et al., 2011).

A potential limitation of our experimental design is that it does not allow a full differentiation of effects on perceptual decision from those on motor execution. Thus, it is possible in principle that the behavioural biases in this study may originate primarily from action selection processes. This is in line with evidence that changes in the oscillatory activity of pre-motor areas are associated with the motor effectors used for the choice (de Lange et al., 2013;Kelly et al., 2021), linking the effect of prior information to increases in motor preparation. It is, however, worth noting that we found no effect of prior probability on the non-decision time parameter of our model, which would be likely to capture differences in motor execution and action selection processes. Additionally, our response-locked analysis (Supplementary Fig. 2) identified a choice-discriminating EEG component with virtually the same topography as the late stimulus-locked EEG component. This finding reinforces the notion that the late EEG component initially starts as stimulus-locked and gradually becomes response-locked, presumably tracking the process of evidence accumulation. Future experiments disentangling the decision from its execution will be required to further discriminate between the perceptual and motor components of the prior probability influences on perceptual choices (see, e.g.,Charlton & Goris, 2024). Another recent study (Feuerriegel et al., 2021) suggested that what determines a starting point or drift rate change may depend on the stimulus presentation duration relative to the length of the temporal integration window, with stimuli presented for a time shorter than that available for recognising them leading to drift rate changes. Our results are consistent with such pre-activation-based accounts as our integration window greatly exceeded the stimulus presentation and therefore the hypothesised pre-activated sensory representations might boost the accumulation process after the stimulus disappears.

It is important to note that, in this rapid visual categorisation task, the evidence entering the decision process likely reflects higher order perceptual representations of the stimulus which are known to persist well after the stimulus disappears and until a choice is made, allowing for object recognition to emerge (see for instance,Franzen et al., 2020;Philiastides & Sajda, 2006,^2007^;Ratcliff et al., 2009). In other words, persistent activity in perceptual areas—likely via local reverberations/memory loops (Coltheart, 1980;Di Lollo, 1977;VanRullen & Koch, 2003)—provides a stream of internal information for the decision areas to accumulate even after the stimulus disappears from the screen.

Consistent with these extended internal dynamics of perceptual processing, the Early component is likely to originate in areas involved in early visual processing and object/face recognition, while the late EEG component is likely to originate in higher-level visual areas generating internal representations of decision-related evidence entering the process of evidence accumulation in parietal and/or frontal cortex (Franzen et al., 2020;Philiastides & Sajda, 2006).

A plausible hypothesis regarding stimulus duration would be that longer stimulus presentation (which would also increase the salience of the stimulus evidence) would trump the post-stimulus amplification effect of the cue. However, preliminary investigations on the same task with longer stimuli (up to 300 ms) identified the same two EEG components. Future work combining EEG and fMRI might ascertain with more confidence the neural origin of the impact of prior probability on these components and by extension on the process of evidence accumulation for the decision. Overall, further investigations employing different decision-making tasks and varying the properties of the presented stimuli will be useful to assess the generalisability of our findings across contexts.

Stimulus-locked EEG features informing our HDDM derive from neural activity that forms the input of the decision process; they do not capture the whole process of evidence accumulation. Our single-trial discrimination analysis was designed to discriminate face from car stimuli (i.e., the stimulus evidence entering the decision), rather than the actual accumulation process that would have been more sensitive to discriminating easy-versus-difficult trials, due to differences in the rate of integration itself. Consequently, the HDDM identifies a change in drift rate and not in starting point, which is unlikely due to our choice of discrimination. Supporting our findings, control analysis for other EEG components from discrimination analysis based on the identity of the cue, rather than the stimuli, which could be sensitive to a starting point bias, did not reveal any significant discriminating power in EEG activity (Supplementary Fig. 6).

In conclusion, our findings suggest that in a visual decision-making task requiring object recognition, prior probability biases are related to increases in the efficiency of information processing leading to the most likely stimulus rather than changes in the baseline activation. Additionally, our work serves as further validation of the importance of using neural signals to inform behavioural models, not only for yielding better parameter estimation and hence more accurate model fits but also for providing novel new insights into the neural underpinnings of behavioural choice that would otherwise be missed or misconstrued by standard (behaviour-only) models.

## Supplementary Material

Supplementary Material

## Data Availability

Data are freely available onhttps://osf.io/xs46h/or can be provided by the corresponding authors upon reasonable request.
