# Supplementary material for: Prior probability biases perceptual choices by modulating the accumulation rate, rather than the baseline, of decision evidence
Source: Imaging Neurosci (Camb). 2024 Nov 18;2:imag-2-00338. doi: 10.1162/imag_a_00338 (PMC12290597; doi:10.1162/imag_a_00338)
Supplement: Supplementary Material [file imag_a_00338-supp.pdf]

Supplementary Material

a

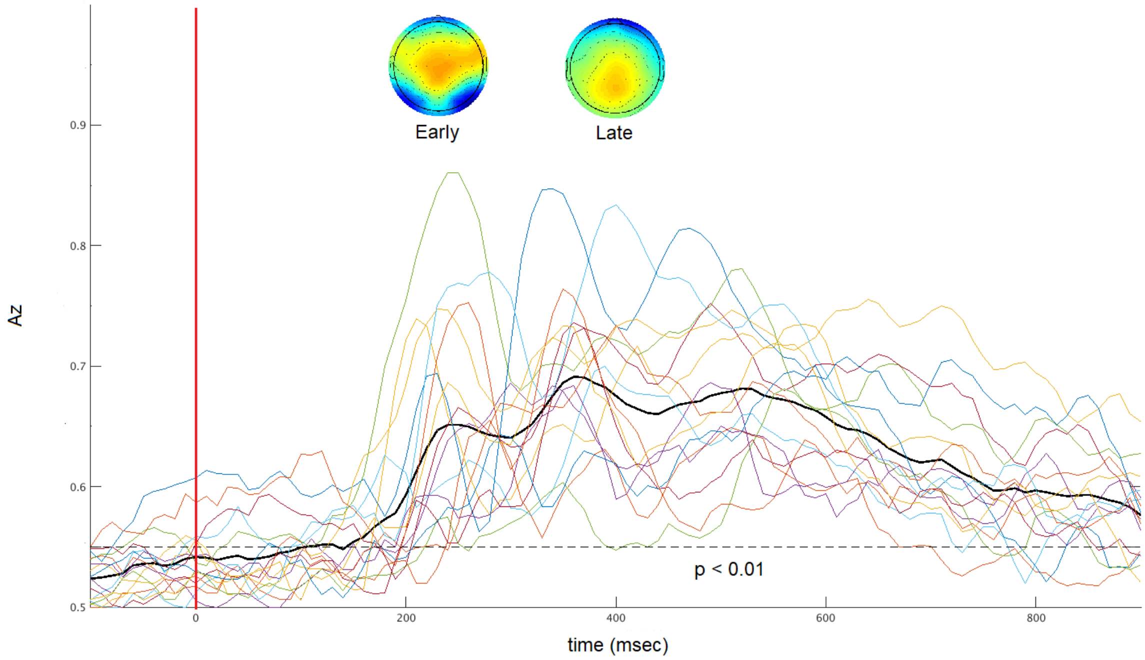

b

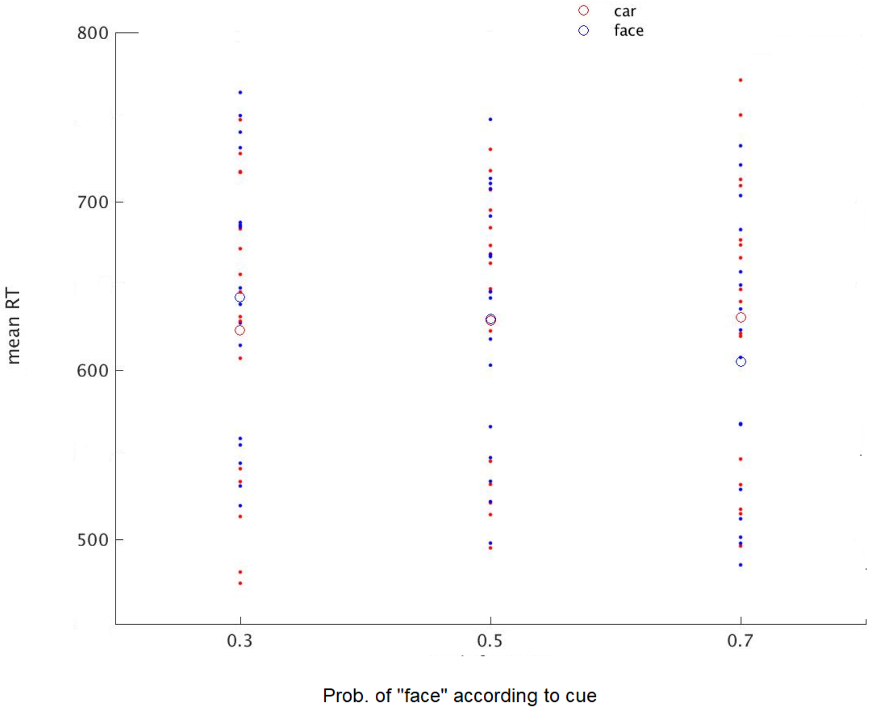

Supplementary Figure 1: Single-trial EEG components. a) Multivariate single-trial discriminator performance (A_z) during face versus car discrimination on stimulus-locked EEG data selected to match the RT distributions in each cue condition, averaged across participants and sessions, showing the presence of an Early and Late component. Faint lines represent individual participant data. In the insets the topography of the two components, representing which electrodes carry more weight for the discriminating component, that is, more discriminating power. The plots represent population averages of the forward model (equation 3) of each participant at peak time. b) Selecting trials to match RT distributions: average reaction time as a function of stimulus probability for the two stimulus types (face: blue, car: red) in a subset of trials which excluded the 8% slowest car trials and the 8% fastest face trials for each of the three cue conditions. Filled dots are individual participant responses. The average RT was matched between face and car trials in the neutral cue condition, whereas participants were faster on the category corresponding to the cue in the other two (i.e., faster for faces in face-cued trials, and vice versa for car-cued).

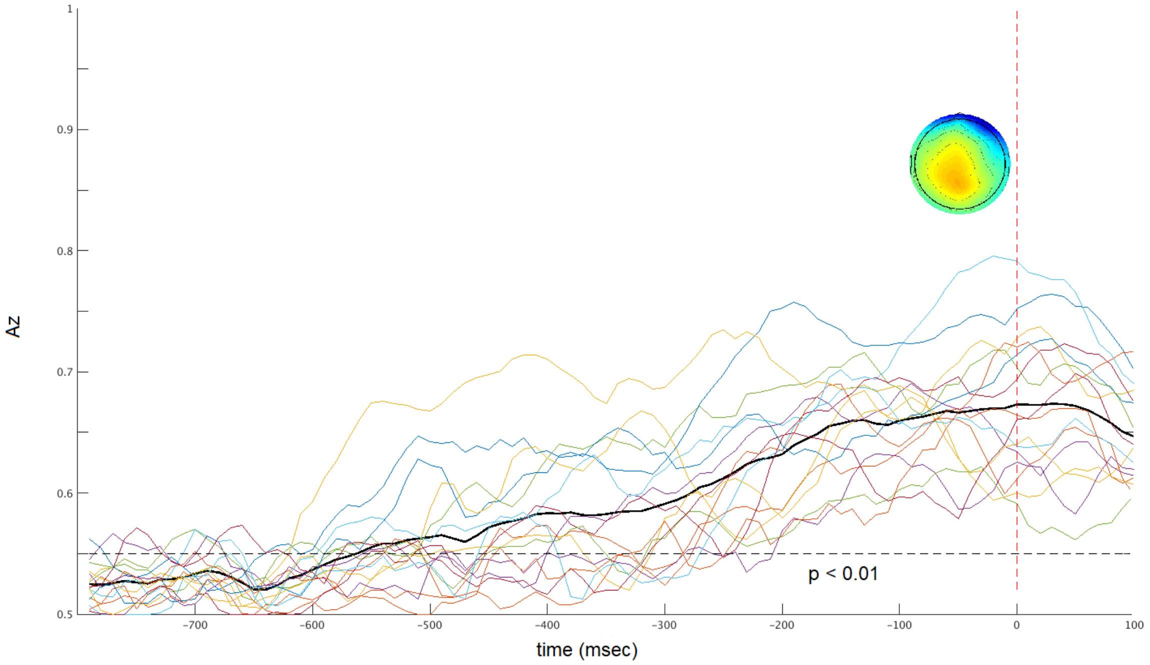

Supplementary Figure 2: Single-trial EEG components. a) Multivariate single-trial discriminator performance (A_z) during face versus car discrimination on response-locked EEG data, averaged across participants and sessions, showing the presence of a response component similar to the Late component in stim-triggered data. Faint lines represent individual participant data. In the inset the topography of the response components, representing which electrodes carry more weight for the discriminating component, that is, more discriminating power. The plot represents the population average of the forward model (equation 3) of each participant at peak time.

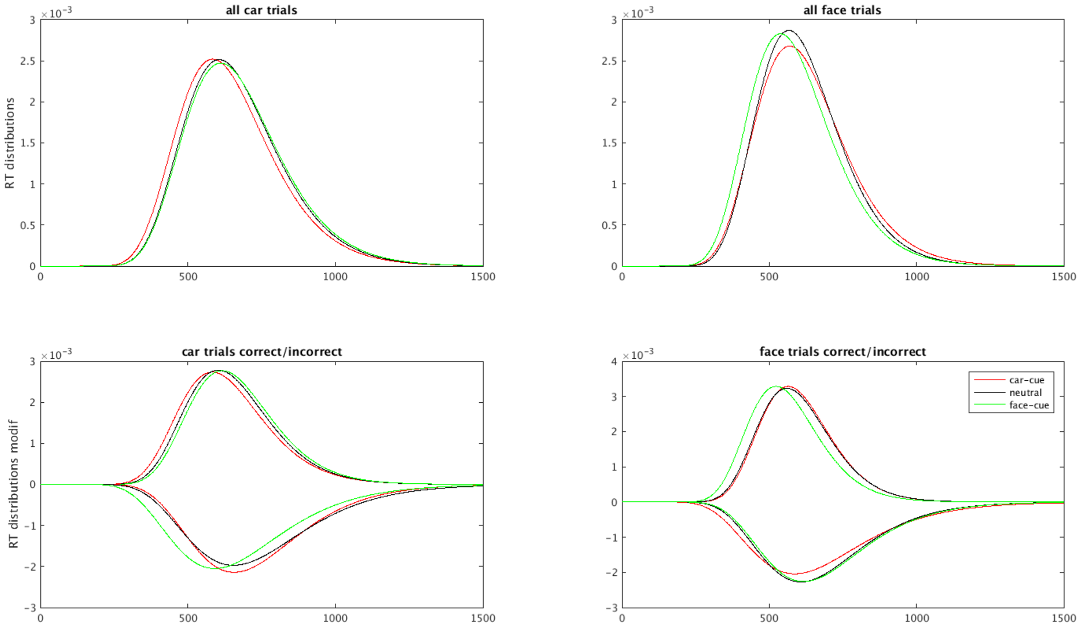

Supplementary Figure 3: RT distributions for car trials (left column) and face trials (right column) divided by cue (car-cue: red, neutral: black, face-cue: green). In the top row for all trials. In the bottom row for correct (positive values) and incorrect (negative values) trials separately.

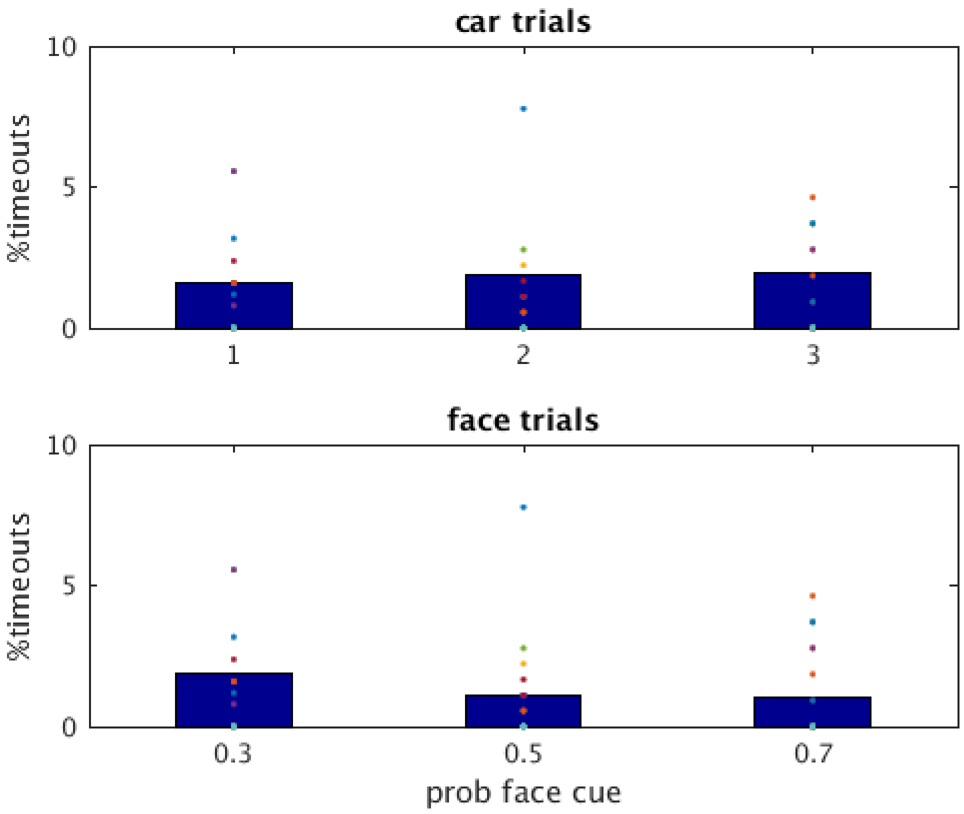

Supplementary Figure 4: The average number of timeouts was low throughout the experiment with less than a handful of trials per condition. On average the frequency of timeouts was 0.016 ± 0.005 with a slight non-significant increase for trials in with unexpected stimuli (face trials with car cues and car trials with face cues) compared to expected ones (0.0191 ± 0.0068 vs 0.0130 ± 0.0043). This effect was stronger for face trials (0.0185 ± 0.0068 vs 0.0104 ± 0.0041) but still not significant ($p = 0.3$). As such we don't expect this difference to drive significant effects on the model fit.

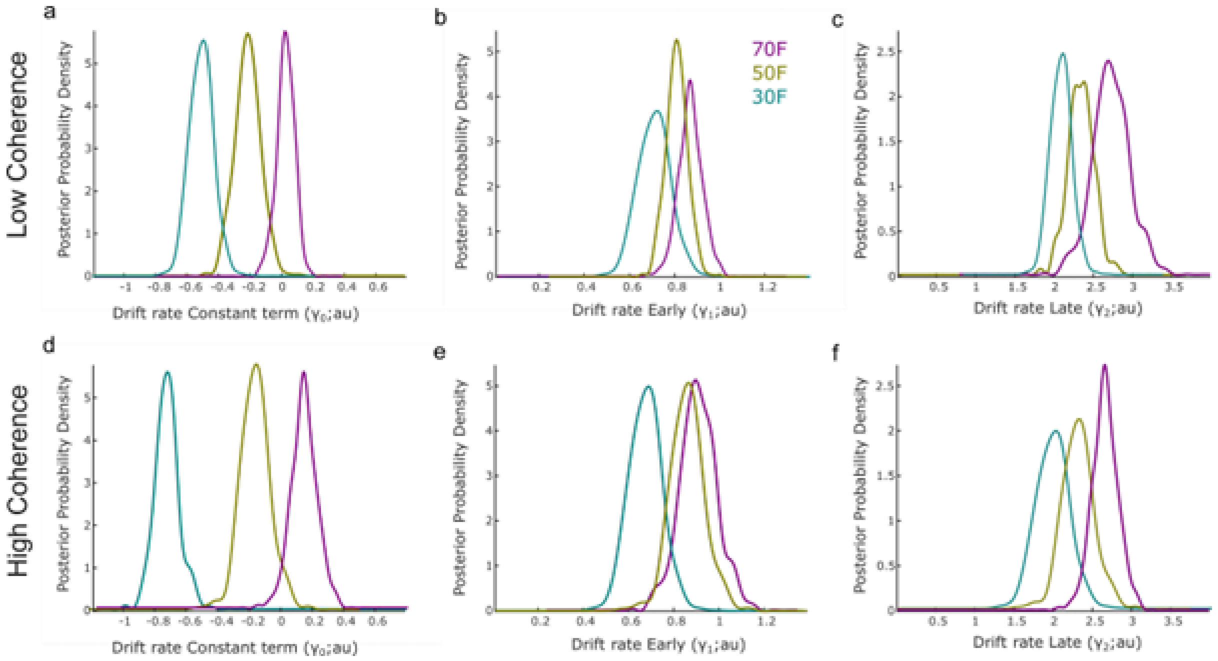

Supplementary Figure 5: nHDDM output parameters estimated separately for the two coherence levels. (a,b). Posterior probability distributions of regression coefficients (γ_0 in a, $\gamma_{1\text{-Early}}$ in b, $\gamma_{2\text{-Late}}$ in d) as predictors of the drift rate (δ) estimated by the nHDDM in low coherence trials for the 70% probability of face (70F; pink), 50% probability of face (50F; yellow) and 30% probability of face (30F; blue) stimulus

probability cues. (c,d,e) same for high coherence trials. Coefficients were derived from the nHDDM including n = 16 independent participants and 17280 trials (split by coherence levels). We found qualitatively similar results to figure 4 (slightly more pronounced for the high-coherence trials).

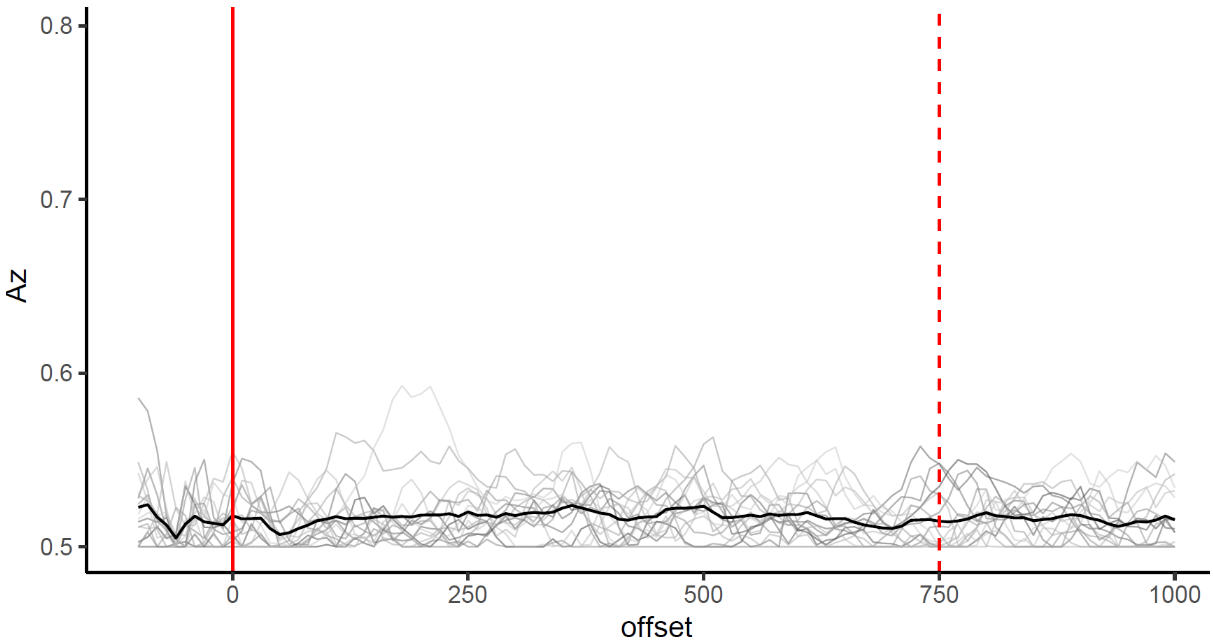

Supplementary Figure 6: Single-trial EEG components. a) Multivariate single-trial discriminator performance (A_z) discriminating cues indicating a face (70% face probability) vs cues indicating a car (70% car probability) on cue-locked EEG data, averaged across participants and sessions. We did not find any significant discriminating power in EEG activity in this post-cue and pre-stimulus time window. The plots represent population averages of the forward model (equation 3) of each participant at peak time.
